# Research progress of molecular typing and targeted therapy for triple-negative breast cancer

**DOI:** 10.3389/fonc.2025.1666126

**Published:** 2025-09-05

**Authors:** Da lin Xiang, Xin Yue

**Affiliations:** Department of Breast Surger, The First Affiliated Hospital of Yangtze University, Jing Zhou, China

**Keywords:** triple-negative breast cancer, high heterogeneity, strong invasiveness, molecular typing, targeted therapy

## Abstract

Triple-negative breast cancer (TNBC) is difficult to treat due to its high heterogeneity, strong invasiveness, high risk of recurrence and metastasis, and poor prognosis. Chemotherapy is still its main treatment, but limited by the lack of specific targets, drug resistance and other factors, the conventional efficacy is poor. In recent years, advances in genomics and other technologies have promoted the deepening of molecular typing studies and the development of targeted therapy for TNBC, providing a new direction for breaking through the therapeutic dilemma. Based on the biological characteristics of TNBC and the current treatment status, this article systematically reviews the latest progress of its molecular typing system, and discusses the breakthrough research results of targeted therapy strategies, aiming to provide a theoretical basis and practical reference for the precise treatment of TNBC.

## 1 Introduction

Triple-negative breast cancer (TNBC) is a subtype of breast cancer that lacks the expression of estrogen receptors (ER), progesterone receptors (PR), and human epidermal growth factor receptor 2 (HER2), and accounts for 15%-20% of all breast cancers (1, 2). Its heterogeneity, high malignancy, youthfulness, easy recurrence and metastasis, and poor prognosis make it a major challenge for clinical treatment (2, 3).

TNBC is ineffective for traditional endocrine therapy and targeted therapy, and at this stage, chemotherapy is the mainstay of treatment, and the adjuvant and neoadjuvant treatment of early TNBC still adopts chemotherapy regimens based on paclitaxel and anthracycline (4). Platinum-containing regimens have been one of the most studied and effective regimens for TNBC patients in recent years. In young patients, especially those with BRCA mutations, the combination of platinum-based regimens with purple shirts has been shown to be highly effective, significantly improving the rate of pathologic complete response (pCR) (5). In addition, there are numerous subtypes of TNBC, and different subtypes have different molecular characteristics, prognoses, and treatment responses. To improve the survival prognosis of TNBC patients, in recent years, researchers have gradually explored the heterogeneity and molecular features of TNBC, and research and clinical trials of related targeted drugs have been conducted one after another, which has led to a major breakthrough in the treatment of TNBC. Based on the existing molecular typing and targeted therapy for TNBC, this article reviews the relevant molecular typing and targeted therapy of TNBC to provide ideas for the precise and individualized treatment of TNBC.

## 2 Molecular typing of TNBC

With the development of genomics, transcriptomics, and metabolomics, increasing attention has been paid to understanding the heterogeneity of breast cancer, and the heterogeneity of TNBC has been widely recognized (6). TNBC is not a single tumor, but a class of highly heterogeneous breast tumors, and there are significant differences in the biological behavior of the tumors and their sensitivity to drugs in different patients (7). Analyzing the intrinsic characteristics of TNBC and distinguishing different molecular types within it is of great significance in guiding the treatment and prognosis of TNBC patients.

### 2.1 Lehmann typing

**Figure 1:**
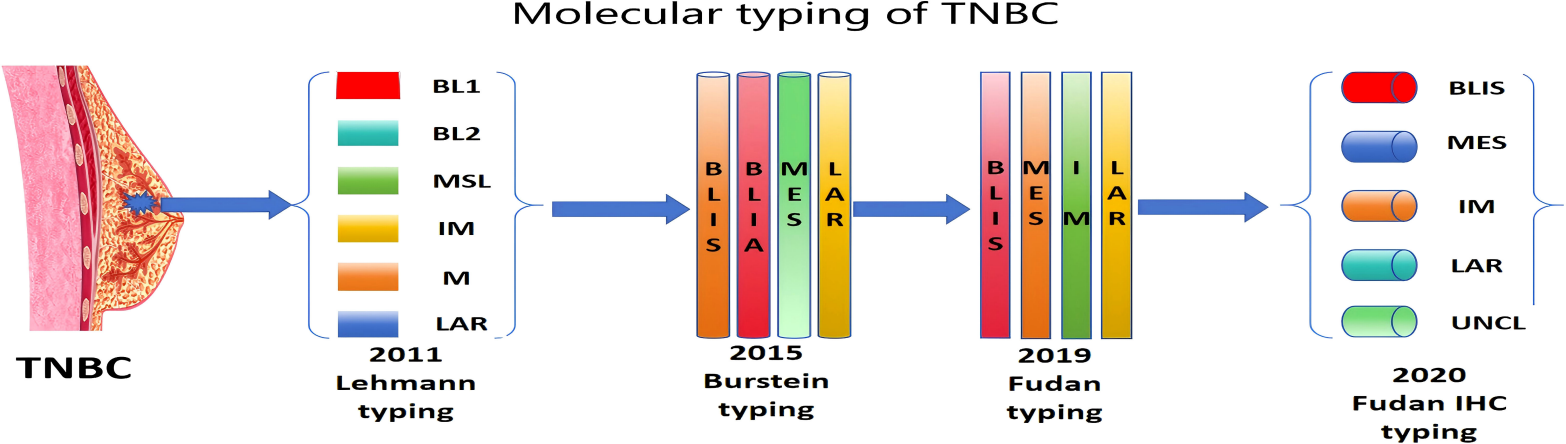
Molecular typing of TNBC; BL1, basal-like 1; BL2, basal-like 2; IM, immunoregulatory; M, mesenchymal; MSL, mesenchymal stem-like; and LAR, luminal androgen receptor; BLIS, basal-like immunosuppressed; BLIA, basal-like immune-activated; MES, mesenchymal; UNCL, unclassifiable.

In 2011, Lehmann et al. (8) combined the information of 587 TNBC patients and proposed for the first time a six-part typing of TNBC based on gene expression profiling, which included basal-like 1 (BL1), basal-like 2 (BL2), immunoregulatory (IM), mesenchymal (M), mesenchymal stem-like (MSL), and luminal androgen receptor (LAR) (**Figure 1**). Each subtype has a different mechanism and corresponding therapeutic regimen as well as therapeutic efficacy. Among them, BL1 and BL2 subtypes have high expression of cell cycle-related genes, active DNA damage repair, and high expression of Ki67, which are more likely to benefit from platinum-based chemotherapy regimens, Possible therapeutic agents for the BL1 subtype include poly (ADP-ribose) polymerase (PARP) inhibitors and genotoxic agents, patients with the BL1 subtype are sensitive to cisplatin therapy, and potentially targeted therapeutic agents for the BL2 subtype include mTOR inhibitors and growth factor inhibitors (lapatinib, gefitinib, and cetuximab) (7); IM subtype is significantly enriched in immune cell-related genes and signal transduction pathways, and IM subtype is highly similar to medullary carcinoma of the breast. Patients with IM subtype breast cancer can be treated with immune checkpoint inhibitors such as PD-1, PDL1, CTLA-4, etc., and the prognosis may be relatively better (7); M and MSL subtypes are enriched in genes related to the epithelial-mesenchymal transition, and M subtypes are characterized by sarcomatous or squamous epithelial cell-like tissues, and are easily resistant to chemotherapy drugs. M subtype has sarcoma-like or squamous epithelial cell-like tissue characteristics and is prone to resistance to chemotherapeutic drugs, and can be treated with mTOR inhibitors or drugs targeting epithelial-mesenchymal transition; compared with M subtype, MSL subtype expresses lower levels of genes related to cell proliferation and higher levels of genes related to stem cells, and patients with MSL subtype can be presumed to be treated with PI3K inhibitors, Src antagonists, or anti-angiogenic drugs, which have been reported in the study Abl/Src inhibitor dasatinib can be used to treat patients with M and MSL breast cancer (7); and the LAR subtype is characterized by high expression of androgen receptor (AR) and active hormone-related pathways, which is ineffective in response to conventional chemotherapy and has unique sensitivity to bicalutamide (an AR antagonist) (7).

Follow-up studies have found the clinical utility of this typing. For neoadjuvant therapy, the BL1 subtype has the highest pCR rate (52%) and the BL2 and AR subtypes have the lowest pCR rates (0% and 10%, respectively), and this subtype is a better predictor of pCR status than the PAM50 intrinsic subtype (9). In 2016, Lehmann et al. further re-updated the typing system using histopathologic quantification and laser capture microdissection, and found that the transcripts of IM and MSL subtypes were derived from lymphocytes and mesenchymal stromal cells, respectively, and therefore integrated IM and MSL subtypes into M subtypes, thus redefining the new TNBC quadruple phenotypes of BL1, BL2, M, and LAR. meanwhile. Comparison of the four subtypes at the clinicopathological level revealed differences in age at diagnosis, histologic grading, local recurrence, distant disease progression, and histopathology (10). Some studies based on Lehmann typing are available and retrospectively analyzed, but there are no targeted prospective clinical trials for subsequent validation.

### 2.2 Burstein typing

On the basis of Lehmann’s typing, some scholars invested in the continued typing and validation of TNBC, and proposed corresponding therapeutic regimens based on the typing.In 2015, Burstein et al (11),from Baylor College of Medicine reclassified TNBC into four subtypes by analyzing the mRNA expression and DNA profiles of 198 cases of TNBC tissues, which were basal-like immunosuppressed (BLIS), basal-like immune-activated (BLIA), mesenchymal (MES), and LAR (11) (**Figure 1**). LAR and MES tumors down-regulate cell cycle regulators and DNA repair genes, whereas MES and BLIA tumors upregulate immune design and immune-related death pathways, BLIS and BLIA subtypes are relatively devoid of P53-dependent gene activation, whereas BLIA tumors highly express and activate STAT genes. These datasets were also used to independently analyze DNA copy number, disease-free survival (DFS), and disease-specific survival (DSS), with BLIA having the best prognosis, and BLIS had the worst prognosis, and to identify genes specific to each subtype, with CCND1 and FGFR2 genes amplified in LAR tumors, while MAGOHB was more frequently amplified in MES, BLIS, and BLIA tumors (11). In contrast, CDK1 was amplified in all four TNBC subtypes (most frequently in BLIA tumors). In the LAR subtype, androgen receptor AR and cell surface mucin 1 are specific targets, and growth factor receptor-related proteins are enriched in the MES subtype. The immunosuppressive molecule VTCN1 is highly expressed in BLIS, and STAT signaling molecules and cytokines are enriched in BLIA (11). Burstein typing not only deepens the typing system, but also proposes the corresponding therapeutic targets based on each subtype. Burstein typing not only deepens the typing system but also proposes corresponding therapeutic targets based on each type of typing, which provides strong evidence for the precise treatment of TNBC based on molecular typing.

### 2.3 Fudan typing

In 2019, Professor Zhimin Shao’s team at the Affiliated Cancer Hospital of Fudan University, through an integrated analysis of genomic, transcriptomic, and clinical data of 465 patients with primary TNBC, classified TNBC into four subtypes: luminal androgen receptor (LAR), basal-like immunosuppressive (BLIS), mesenchymal (MES), and immune-modulated (IM) (12) (**Figure 1**). By parsing the molecular biology and clinicopathological features of different subtypes, potential therapeutic targets based on Fudan typing have been proposed: the LAR subtype relies on the AR signaling pathway and is often accompanied by mutations in the HER-2 pathway, CDK4/6 inhibitors with AR inhibitors are possible therapeutic targets, and for HER-2 mutations, anti-HER-2 targeted therapy can be attempted. The IM subtype has abundant immune cell infiltration, the MES subtype is relatively enriched in tumor stem cells and PI3K/AKT-related pathway genes, and mTOR inhibitors may be used for the treatment of patients with mutations in this subtype. The BLIS subtype has a relatively poor prognosis and is associated with DNA damage repair defects, which are often accompanied by BRCA1/2 mutations or homologous recombination deficiency (HER-2). Homologous recombination deficiency (HRD). Based on the HRD score, BLIS can be further subdivided into high- and low-HRD subtypes, with patients in the high-HRD subtype being more likely to benefit from platinum-based chemotherapeutic agents. To validate the significance of this subtype for clinical treatment, a translational study was conducted in 2023 in the center of 141 advanced breast cancers by subtype (13), which were divided into seven groups according to the subtype and mutation for the corresponding clinical treatments; 42 patients achieved an objective response, with a median time to response of 1.8 months and a median duration of response of 4.9 months. Nine patients had a long-term response time of > 12 months,and 68 patients had a long-term response time of > 12 months. times more than 12 months and 68 patients achieved disease control. The strategy guided by this subtype analysis and genomic sequencing has good efficacy and manageable toxicity in patients with metastatic TNBC, and can be gradually translated into clinical treatment.

Due to the long time and high cost of genetic and transcriptional analysis research, as well as the high requirements for subsequent data analysis and interpretation, Lehmann and Burstein typing is still in the research and exploration stage and has not yet been directly used for clinical decision-making. Based on this, the Fudan team proposed the use of immunohistochemistry as an alternative. By analyzing the TNBC RNA sequencing data from the Fudan University Shanghai Cancer Center (FUSCC) (n=360) and The Cancer Genome Atlas dataset (n=158), we screened four immunohistochemistries that are commonly used in clinical immunohistochemistry (IHC), namely AR, FOXC1, CD8, and DCLK1, which were classified into five types based on immunohistochemistry: IM (AR-CD8+), LAR (AR+), BLIS (AR-CD8-FOXC1+), MES (AR-CD8-FOXC1-DCLK1+), and unclassifiable(AR-CD8-FOXC1-DCLK1-)) (**Figure 1**). External validation was performed, and it was found that the results of being able to use immunohistochemistry to quickly and conveniently determine the FUSCC quadruple typing in TNBC patients were highly consistent with the typing derived from the results of genetic testing. Thus, the typing has a strong clinical promotion value (14), and a number of hospitals and centers have been gradually carrying out the corresponding immunohistochemical tests and applying them to the clinic.

Molecular typing of TNBC is the cornerstone for achieving precision targeted therapy, but existing studies still suffer from insufficient sample size, high cost and difficult clinical application. In the future, it is necessary to achieve more accurate molecular typing on the basis of the existing quadruple typing through larger multicentre cohort and multidimensional studies, and to develop targeted drugs (15), so as to achieve precise and individualized treatment. At present, the immunohistochemical typing proposed by Fudan University has more prospects for clinical promotion due to its easy operation and low cost.

## 3 Targeted therapy for TNBC

### 3.1 Poly(adenosine diphosphate ribose) polymerase inhibitors

PARPi is a class of targeted drugs that exert antitumor effects by blocking the DNA repair function of PARP (poly(adenosine diphosphate) ribose polymerase), a class of therapeutic drugs directed against PARP proteins involved in DNA repair, to which cancer cells with homologous recombination repair defects (especially BRCA variants) have increased sensitivity, inducing synthetic lethality and exerting antitumor effects (16). Those that have been approved for tumor therapy include olaparib, talazoparib, rucaparib, and niraparib (16, 17). Currently, olaparib is approved for BRCA1/2 mutated HER2-negative metastatic breast cancer, and talazoparib is approved for BRCA1/2 mutated advanced breast cancer. Niraparib and rucaparib are mainly used in the treatment of ovarian cancer, but several combination therapy trials in breast cancer have been conducted in combination therapy trials (18), and niraparib in combination with anti-angiogenic drugs has been successful in mouse models and may be applied to BRCA wild-type patients at a later stage (19). In the phase 3 OlympiAD trial (20), olaparib improved progression-free survival (PFS) and doubled the objective response rate (ORR) in patients with metastatic breast cancer. An extended follow-up analysis showed that overall survival (OS) was significantly longer in patients treated with olaparib as first-line therapy than in the standard chemotherapy group. Another phase 3 OlympiA trial (21) evaluated 1-year olaparib versus placebo in patients with high-risk HER-2 negative breast cancer harboring the gBRCA1/2 mutation after chemotherapy, and olaparib reduced the risk of recurrence (3-year invasive disease-free survival, 85.9% vs. 77.1%; 3-year distant disease-free survival, 87.5% vs. 80.4%) and reduced the risk of death by approximately 30%. In the phase 3 EMBRACA trial (22), talazoparib improved PFS and ORR in gBRCA1/2 variant carriers pretreated with up to three chemotherapeutic regimens, but talazoparib did not improve OS. In both trials, a significant improvement in health-related quality of life was observed in patients treated with PARP inhibitors and delayed time to progression was observed in patients treated with PARP inhibitors. In the NEOTALA phase 2 trial (23), neoadjuvant talazoparib for 24 weeks resulted in a pCR of 53% in TNBC carriers of the gBRCA1/2 variant, which is similar to the results achieved with neoadjuvant regimens of conventional chemotherapy with fewer side effects. Some small studies have suggested that PARPi in combination with immunotherapy and antibody-drug conjugates (ADCs) may increase treatment efficacy. Although PARPi has been gradually applied in the clinic, drug resistance is more common, and some side effects are more serious. In the future, it will be necessary to further elaborate on the resistance mechanism and explore the combination of PARPi with other drugs to overcome drug resistance. For example, high expression of FANCI is associated with PARP inhibitor resistance, and inhibition of FANCI can restore sensitivity (24).

### 3.2 PI3K/AKT/mTOR signaling pathway inhibitors

The PI3K/AKT/mTOR pathway is frequently mutated or over-activated (e.g., PIK3CA, AKT1, or PTEN gene alterations) in TNBC, and abnormalities in this pathway are present in approximately 70% of patients with TNBC (25). This pathway promotes TNBC progression by regulating cell proliferation, survival, metabolism, and chemoresistance,and over-activation of this pathway is associated with aggressiveness, high recurrence rates, and poor prognosis in TNBC (25, 26). Mutations in the PI3KCA gene contribute to tumorigenesis, and studies have demonstrated that PI3KCA is mutated in 20-40% of breast cancers and has been associated with increased chemoresistance, with approximately 10% of TNBC having PI3KCA mutations, but they are more common in LAR and MES subtypes (3). To date, a variety of PI3K/AKT/mTOR signaling pathway-targeted drugs have been developed and utilized in clinical trials for the treatment of TNBC, and most of them have demonstrated good therapeutic efficacy when combined with other chemotherapeutic agents or targeted drugs It has been reported that common PI3K/AKT/mTOR pathway inhibitors include apelisib, ipatasertib, capivasertib and everolimus, etc. (3). The LOTUS and PAKT trials demonstrated (27, 28), respectively, that the addition of the AKT inhibitors ipatasertib or capivasertib to the first-line paclitaxel treatment of metastatic TNBC significantly prolonged PFS, and the clinical benefit was more pronounced in patients harboring PIK3CA/AKT1/PTEN mutations. The FAIRLANE study also evaluated the efficacy of neoadjuvant ipatasertib in combination with paclitaxel for the treatment of early-stage TNBC, and the ipatasertib treatment group had a higher pCR than the placebo-treated group in patients with mutations in the PI3K/AKT/mTOR signaling pathway (27, 28). The pCR was higher than that in the placebo group (29). Everolimus, an oral mTOR inhibitor, has been shown to be effective when added to eribulin treatment for metastatic breast cancer, and everolimus in combination with cisplatin is effective in TNBC patients with residual lesions after standard neoadjuvant chemotherapy. In conclusion, a number of preclinical evidence confirms that PI3K/AKT/mTOR inhibitors have good efficacy in TNBC, but single-agent efficacy is limited, easy to drug resistance, the later still need to continue to explore the use of chemotherapy with chemotherapy, multi-targeted drugs in combination and other treatments.

### 3.3 Angiogenesis inhibitors

Vascular endothelial growth factor (VEGF) binding to its corresponding receptor can promote tumor angiogenesis, increase vascular permeability, and promote tumor proliferation and metastasis (30). Studies have shown that the expression level of VEGF in TNBC patients (especially BLIS type) is significantly higher than that in non-TNBC, patients, so anti-angiogenic drugs can effectively stop tumor development, commonly used bevacizumab,apatinib, etc. (3, 14). The RIBBON1 phase III trial showed that bevacizumab was significantly more effective than the conventional capecitabine, anthracycline, or paclitaxel combination in improving PFS in patients with mTNBC. mPFS was prolonged in the bevacizumab-treated group compared with the placebo-treated group (6.0m vs. 2.7m), and there was a trend toward improved OS in patients with mTNBC (3). The GeparSixto and GeparQunito trials combined bevacizumab with neoadjuvant chemotherapy for the treatment of TNBC patients and showed a significant improvement in pCR in TNBC patients. However, the results of the BEATRICE trial showed that bevacizumab failed to improve OS in early stage TNBC patients, as well as higher side effects of bevacizumab and inconsistency in the treatment of bevacizumab TNBC patients, and the FDA withdrew bevacizumab from breast cancer treatment (3). aptinib was shown to be effective in treating breast cancer through inhibition of the TNBC cellular vascular endothelial growth factor receptor (VEGFR) signaling, thereby inhibiting tumor growth.The NAN trial demonstrated (31) that the addition of apatinib to advanced TNBC patients who had failed first-/second-line therapies improved their PFS and had a favorable safety profile.Liu et al. verified that the combination of camrelizumab and apatinib effectively improved the ORR of advanced ORR in patients with TNBC (32). In patients with advanced TNBC who failed at least one line of chemotherapy, the median PFS and overall survival (OS) of apatinib in combination with etoposide were significantly better than those in the chemotherapy-only group (PFS: 50.0% vs. 6.7%; OS: 90.0% vs. 20.0%) (33). Tumor angiogenesis is a complex process, and a series of molecular mechanisms and precise target studies are needed to support the realization of tumor treatment through anti-angiogenesis as well as further clinical trials to evaluate the effects and adverse reactions of anti-angiogenic therapy.

### 3.4 AR inhibitors

The expression rate of AR in TNBC is 12%-55%, of which the LAR subtype is a unique subtype of TNBC, accounting for 15% of TNBC. The LAR subtype highly expresses AR and AR target genes, and the level of AR expression in the LAR is negatively correlated with the PFS and OS of TNBC patients (34). Currently, common AR inhibitors include enzalutamide, bicalutamide, GTx-024, etc. (3). The TBCRC011 phase II clinical study (35) enrolled 51 AR-positive, ER/PR-negative patients with advanced breast cancer to analyze oral bicalutamide for more than 6 months, and the results showed a clinical benefit rate (CBR) of 19%, a median PFS of 12 weeks, and good tolerability. Another study showed (36) that in AR+ TNBC, the 6-month CBR of oral bicalutamide (used in 30% of patients) was 29% and well tolerated. Enzalutamide is a second-generation nonsteroidal AR inhibitor with a stronger inhibitory activity than bicalutamide. enzalutamide showed good clinical efficacy and tolerability in patients with AR+ TNBC, with mPFS and mOS of 3.3 and 17.6 months, respectively, and a 2% incidence of serious adverse events in patients (3). However, its performance in the FUTURE trial (14) in combination with CDK4/6 inhibitors failed to show good efficacy in patients with TNBC who had failed multiple lines of therapy. Enzalutamide and bicalutamide showed some clinical activity in AR+ TNBC but had limited efficacy as single agents. Current research focuses is shifting towards combination therapy (chemotherapy, targeted agents, or immunotherapy) and biomarker-guided therapies.

### 3.5 Antibody–drug conjugate

ADC drugs are a rapidly developing class of antitumor drugs, which are mainly composed of antibody carriers, cytotoxic drugs, and linkage junctions. They mainly use antibodies as carriers to deliver cytotoxic drugs into the tumor cells, which damage the double-stranded DNA and further lead to the death of the tumor cells, resulting in high tolerance and enhanced cytotoxicity. Currently, there are two main classes of ADC drugs in TNBC therapy: targeting trophoblast cell surface antigen 2 (Trop-2) and HER2.

#### 3.5.1 Trop-2 ADC

Trop-2 is a transmembrane glycoprotein that is significantly overexpressed in TNBC (with a higher incidence than other breast cancer subtypes), and plays a key role in tumor growth and leads to a more aggressive and poorer prognosis (37). Sacituzumab Govitecan (SG) is the first globally approved Trop-2-targeted drug for the treatment of metastatic TNBC ADC, and consists of an anti-Trop-2 monoclonal antibody coupled with the topoisomerase I inhibitor SN-38. The phase III ASCENT trial confirmed (38) that SG significantly improved ORR (35% vs. 5%), PFS (5.6m vs. 1.7m), and OS (12.1 m vs. 6.7m) in patients with mTNBC compared to standard chemotherapy regimens, and that patients with high expression of Trop-2 were more likely to benefit from SG treatment. In addition, ASCENT series of trials demonstrated (39) that chemotherapy-naïve patients with mTNBC had significantly improved PFS and OS after SG treatment. Meanwhile, some of the current to suggest that SG has significant efficacy in the adjuvant treatment of TNBC neoadjuvant or metastatic breast cancer, and also has efficacy in Her-2 negative, ER(+) breast cancer. Datopotamab deruxtecan (Dato-DXd) is another ADC drug targeting Trop-2, which consists of a monoclonal antibody targeting Trop-2, the topoisomerase I inhibitor DXd and a cleavable tetrapeptide linker. The phase I Dato-DXd clinical trial showed (40) a median progression-free survival of 4.4 months and a median sustained remission time of 16.8 months with safe and manageable adverse events in the TNBC cohort treated with Dato-DXd, with no significant benefit in OS at this time, and is currently being evaluated in a phase III study.

#### 3.5.2 HER2 ADS

Trastuzumab Deruxtecan (DS-8201a, T-DXd) is a combination of the HER2-targeting antibody trastuzumab, a tetrapeptide-cleavable linker, and the novel cytotoxic drug DNA topoisomerase I inhibitor deruxtecan (DXd) (41). DS-8201a has a high drug-antibody ratio, high cell membrane permeability, and potent bystander effect, DS-8201a has been approved for the second-line and above treatment of metastatic HER2-positive breast cancer and HER-2 low-expressing breast cancer (IHC 1+ or 2+/ISH-). The DESTINY-Breast04 trial demonstrated, for the first time, that patients with HER-2 low-expressing breast cancers, including TNBC subtypes, could benefit from treatment with DS-8201a, which significantly improved HER-2 expression compared with chemotherapy. Compared with chemotherapy, DS-8201a significantly improved PFS and OS in patients with advanced breast cancer with low HER-2 expression. months and reduced the hormone receptor-negative subgroup, the DS-8201a treatment group prolonged the median PFS by 5.6 months and reduced the risk of disease progression or death by 54%, and the median OS prolonged the median OS by 9.9 months and reduced the risk of death by 52%, compared with the chemotherapy group (41).

### 3.6 CDK inhibitors

The main difference between tumor cells and normal cells is that tumor cells can proliferate wirelessly, CDK is a key enzyme that regulates the transition of cell cycle phases, and its continuous activation can lead to proliferation of tumor cells.CDK4/6 is a key regulator of the cell cycle and phosphorylates RB protein with cyclin D, which triggers the cell cycle to enter the S-phase from the G1-phase, thus inhibiting the cellular DNA replication process. TNBC has a stronger proliferative and invasive ability compared to other subtypes of breast cancer (3). The LAR subtype is highly sensitive to CDK4/6 inhibitors, and the use of CDK4/6 inhibitors may be a potential therapeutic approach for this LAR subtype (42). Currently, commonly used CDK4/6 inhibitor drugs, such as palbociclib, ribociclib, and abemaciclib are approved for first- or second-line treatment of HR+/HER-2- advanced or metastatic breast cancer. TNBC cells segregate CDK4/6 inhibitors due of high lysosomal content, which prevents drugs from reaching their nuclear targets, leading to ineffective treatment and natural resistance (42). Currently, the efficacy of single-agent treatment is poor and the effect of combination therapy with other drugs is significant. In the Rb-positive TNBC cell model, palbociclib and paclitaxel were combined simultaneously, and the combination of treatments was more effective than single treatments in inhibiting cell proliferation and increasing cell death (43). Shao et al. found that the combination of palbociclib and olaparib had synergistic anti-tumor effects on TNBC cells in cell models (44).

### 3.7 Other targeted therapies

As the exploration of the molecular characterization of TNBC continues, a series of new targets with developmental potential have been identified, enriching the scope of targeted TNBC therapy. Most of these new targets are still in preclinical studies and clinical trials, including the Hippo/YAP signaling pathway, MAPK signaling pathway, JAK2/STAT3 pathway, Notch signaling pathway, Src signaling pathway, tumor stem cells, and antibody-small interfering RNA (siRNA) affixes (45). At present, several novel targeted drugs have achieved initial results in clinical trials, and several other drugs are under development, which are expected to be put into the clinic in the future to serve patients and change the landscape of TNBC-targeted therapy. The targeted development of specific targeted drugs and drug combination programs should be attempted to improve efficacy while reducing drug-related adverse reactions in patients.

## 4 Summary and outlook

TNBC is a subtype of breast cancer with high malignancy, easy recurrence and metastasis, and poor prognosis; it is insensitive to endocrine therapy and traditional anti-HER2 targeted therapy, and chemotherapy is the main therapeutic strategy. With the development of genomics and transcriptomics, TNBC molecular typing has gradually penetrated into clinical research and treatment, and targeted therapy, immunotherapy and combination therapy have been gradually carried out in the clinic, and the TNBC molecular typing-oriented treatment strategy is expected to add bricks and mortar to the precise treatment strategy of “classification and treatment.” However, the existing therapeutic options are still limited, and there are still many problems and challenges in improving the efficacy of TNBC, such as comprehensively analyzing the TNBC ecosystem, developing clinically accessible new targets, exploring better drug combinations, and investigating the mechanisms of drug resistance and its resolution. In the future, an in-depth understanding of TNBC heterogeneity is needed, including the characteristics of different tumors and the unique features of each patient, as well as searching for evidence-based medicine and conducting precision clinical trials to further improve the prognosis of TNBC patients.
